# Validation of the Colibrí Instrument for Automated Preparation of MALDI-TOF MS Targets for Yeast Identification

**DOI:** 10.1128/jcm.00237-22

**Published:** 2022-06-15

**Authors:** Robbe Heestermans, Pauline Herroelen, Kristof Emmerechts, Kristof Vandoorslaer, Deborah De Geyter, Thomas Demuyser, Ingrid Wybo, Denis Piérard, Astrid Muyldermans

**Affiliations:** a Vrije Universiteit Brussel (VUB), Universitair Ziekenhuis Brussel (UZ Brussel), Department of Microbiology and Infection Control, Brussels, Belgium; University of Utah

**Keywords:** automation, diagnostics, phenotypic identification, yeasts

## Abstract

Recently, Copan (Italy) introduced the Colibrí instrument for automated colony picking and preparation of matrix-assisted laser desorption ionization–time of flight (MALDI-TOF) target plates. Our study aimed to validate this system for yeasts as such testing has not been performed yet and is a missing link needed to implement the system for routine use. Fifty-five *Candida* strains were selected to evaluate the accuracy of Colibrí. For each strain, a sheep blood agar plate supplemented with X and V factors (HEM) and a Sabouraud agar plate (SAB) were inoculated and incubated using the WASPlab specimen processing system (Copan). After 18 h and 36 h of incubation, the isolates were spotted in parallel using Colibrí and manually onto MALDI-TOF target plates with the addition of formic acid and identified using MALDI-TOF mass spectrometry. The reproducibility was evaluated using ATCC reference and clinical isolate-derived strains. The cumulative percentage of acceptable identification scores (IDs) after 36 h was 91% for strains cultured on HEM plates using both Colibrí and the manual method. The SAB plates showed inferior results for both Colibrí (76%) and the manual method (78%). We observed an overall agreement of 92% at 18 h for identification of the strains on the HEM plates between Colibrí and the manual method and 94% after 36 h. For the SAB plates, the agreement was 78% after 18 h and 84% after 36 h. Apart from Candida dubliniensis and Candida tropicalis, all *Candida* species were identified with 100% accuracy using Colibrí on HEM plates. We observed good agreement between Colibrí and the manual reference method. These results demonstrate that Colibrí is a reliable system for MALDI-TOF target preparation for yeast identification, allowing increased standardization and less hands-on time.

## INTRODUCTION

Invasive fungal infections are a growing source of concern worldwide. The reported incidence in European hospitals is situated between 3 and 8.6 cases per 100,000 patients per year, with a higher incidence observed among young (<1 year) infants, as well as among the elderly (>70 years) (1). The overall mortality risk for patients with candidemia is estimated to range between 10 and 20% (2). While Candida albicans remains the most common causative pathogen, there is an increasing incidence of candidemia caused by non-*albicans* species, with C. glabrata, C. tropicalis, C. parapsilosis, and C. krusei having a growing importance (3). During the past decade, Candida auris has emerged as a novel yeast species that is capable of causing major outbreaks and is associated with a high level of resistance toward commonly used antifungals (4). Therefore, rapid and reliable identification of the causative pathogen is of major importance for guiding therapeutic choices.

To identify these pathogens, most clinical microbiology laboratories nowadays use matrix-assisted laser desorption ionization–time of flight mass spectrometry (MALDI-TOF MS), which enables fast and accurate identification (5). The introduction of this technique has dramatically changed the laboratory workflow during the past decade. However, preparation of MALDI-TOF target plates remains a time-consuming activity which necessitates considerable hands-on time. With ongoing evolution toward more automation in clinical microbiology laboratories, providing better traceability and standardization, Copan (Brescia, Italy) recently introduced the Colibrí instrument. This device can pick colonies from culture plates selected using WASPLab (Copan) and use them for preparation of MALDI-TOF target plates (6). Bielli et al. obtained an agreement of 96.9% and 82.6% between Colibrí and the manual spot-plating method for identification of 33 Gram-negative and 23 Gram-positive bacterium strains, respectively (7). Similarly good results were shown by Paolucci et al., where all Gram-negative uropathogens spot-plated using Colibrí were correctly identified using the MALDI Biotyper (Bruker, Bremen, Germany), and a 100% agreement in identification results was observed between the different culture media used in this study (8). However, the Colibrí system has not yet been validated for yeast identification. In this study, we therefore aim to evaluate the performance of Colibrí for target preparation of yeasts and subsequent identification with MALDI-TOF.

## MATERIALS AND METHODS

### Strains.

Overall, 55 nonduplicate yeast strains were included, consisting of reference strains and strains isolated from clinical samples (blood cultures) stored at −80°C. These included C. albicans (*n* = 7), C. glabrata (*n* = 9), C. parapsilosis (*n* = 7), C. tropicalis (*n* = 8), C. krusei (*n* = 2), C. guilliermondii (*n* = 6), C. dubliniensis (*n* = 9), C. auris (*n* = 2), and C. lusitaniae (*n* = 5).

### Processing of culture plates using WASPLab and Colibrí.

**FIG 1:**
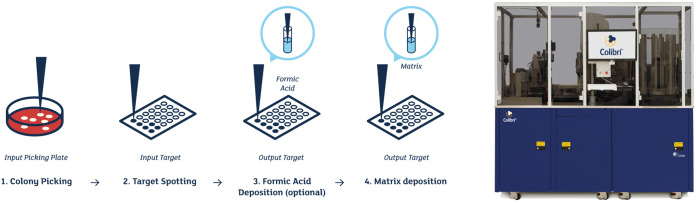
MALDI-TOF target preparation steps using Colibrí. Graphical overview of the Colibrí colony-picking mechanism. (Adopted and modified from reference 9 with permission of the publisher.)

Thawed yeast strains were used for manual inoculation of a sheep blood agar plate supplemented with X and V factors (in-house preparation, henceforth called a HEM plate) and a Sabouraud agar (SAB) plate (Becton, Dickinson and Company, Heidelberg, Germany). These plates were incubated (35°C; 5% CO_2_ for the HEM plates) for 24 h until there was sufficient growth to prepare a 0.5 McFarland suspension. This suspension was used with WASPLab to inoculate a HEM plate and a SAB plate for each yeast strain, which were subsequently incubated (35°C; 5% CO_2_ for the HEM plates) in WASPLab for 36 h. Digital images of the culture plates were assessed using the WASPLab Webapp at predefined time points, i.e., after 18 h and 36 h of incubation. If there was sufficient growth after 18 h of incubation, colonies were selected from the culture plates for picking by Colibrí using the WASPLab Webapp. After processing on WASPLab, the culture plates were loaded into the Colibrí instrument for picking colonies and spotting them onto a reusable MALDI-TOF target plate (Bruker). As illustrated in Fig. 1, the Colibrí pipetting system directly picks the preselected colony and transfers a standard amount of colony material onto the MALDI-TOF target plate. Formic acid and matrix solution are subsequently added automatically onto the spot on the MALDI-TOF target plate by Colibrí. If there was insufficient yeast growth (i.e., absence of targetable individual colonies) after 18 h, the culture plates were reincubated and processed only after 36 h. Manual picking of colonies and spotting them onto a MALDI-TOF target plate were conducted in parallel by the same person using toothpicks. Formic acid and matrix solution were subsequently pipetted manually onto the spot-plated colonies.

### Identification using MALDI-TOF.

The isolates were identified using MALDI-TOF with the Microflex LT mass spectrometer and a MALDI Biotyper Sirius system (Bruker). The MALDI Biotyper CA system has been FDA approved for the identification of all *Candida* species that have been included in this study and is CE-IVD certified (approved by the European Commission for *in vitro* use) as well. MBT Compass software version 4.1.100 and the Bruker Taxonomy database version 9 were used. Identification of yeasts was first conducted automatically in research use only (RUO) mode. The default “spiral_small” pattern of the MALDI Biotyper was used for the automatic acquisition and identification of the spot-plated colonies. With the acceptance criterion for identification on the species level set at a score of ≥1.8 (validated in-house), yeasts with an identification score of <1.8 were reanalyzed on the MALDI-TOF through manual reshooting of the target. The same workup was performed for the manually spot-plated strains.

### Reproducibility.

To evaluate the within-run and between-run reproducibility of Colibrí for yeast identification, the following two ATCC reference strains were used: C. albicans (ATCC 90028) and C. glabrata (ATCC 90030). In addition, the reproducibility was evaluated on C. albicans and C. glabrata strains that had been isolated from clinical samples. The strains were inoculated onto a HEM plate and an SAB plate and processed using WASPLab as described above. To assess the within-run reproducibility, 10 colonies from each plate were selected for picking by Colibrí and identification with MALDI-TOF. To evaluate the between-run reproducibility, one colony from each culture plate was picked by Colibrí and subsequently identified with MALDI-TOF for 10 consecutive days. As was done for evaluation of the accuracy of Colibrí, the data obtained through manual reshooting were included to assess the reproducibility.

### Statistical analysis.

Statistical analyses were performed using Microsoft Excel version 2016 (USA) and MedCalc version 14.8.1 (Belgium). A chi-square test was used to compare the proportions of categorical variables. Differences were considered to be statistically significant if the *P* value was <0.05.

## RESULTS

### Acceptable identification of yeast strains after 18 h and 36 h of incubation.

Sufficient growth after 18 h of incubation was observed for 41/55 HEM and 49/55 SAB plates. All included C. glabrata strains (*n* = 9) showed insufficient growth on HEM plates after 18 h of incubation. Detailed results for the acceptable identification scores (IDs) are shown in Table 1. The percentage of acceptable IDs after 18 h was calculated with the inclusion of manual reshooting data. Strains with insufficient growth after 18 h were not included in the calculations at this time point. Acceptable ID percentages after 36 h were calculated with the inclusion of results obtained after 18 h and additionally identified strains after 36 h of incubation (with the inclusion of manual reshooting data).

**TABLE 1:** Rates of acceptable identification after 18 h and 36 h of incubation (cumulative percentages)^*a*^

Species	% strains identified (no. identified/total no. of strains)							
	18 h				36 h			
	HEM		SAB		HEM		SAB	
	Colibrí	Manual	Colibrí	Manual	Colibrí	Manual	Colibrí	Manual
Candida albicans	100 (6/6)	67 (4/6)	83 (5/6)	100 (6/6)	100 (7/7)	100 (7/7)	71 (5/7)	86 (6/7)
Candida krusei	100 (2/2)	100 (2/2)	100 (2/2)	100 (2/2)	100 (2/2)	100 (2/2)	100 (2/2)	100 (2/2)
Candida auris	100 (2/2)	100 (2/2)	100 (2/2)	100 (2/2)	100 (2/2)	100 (2/2)	100 (2/2)	100 (2/2)
Candida dubliniensis	67 (6/9)	89 (8/9)	22 (2/9)	44 (4/9)	78 (7/9)	89 (8/9)	22 (2/9)	44 (4/9)
Candida guilliermondii	100 (6/6)	100 (6/6)	100 (6/6)	100 (6/6)	100 (6/6)	100 (6/6)	100 (6/6)	100 (6/6)
Candida parapsilosis	100 (3/3)	100 (3/3)	66 (2/3)	100 (3/3)	100 (7/7)	100 (7/7)	86 (6/7)	71 (5/7)
Candida tropicalis	88 (7/8)	100 (8/8)	63 (5/8)	88 (7/8)	88 (7/8)	100 (8/8)	63 (5/8)	88 (7/8)
Candida glabrata ^*b*^			88 (7/8)	63 (5/8)	78 (7/9)	56 (5/9)	100 (9/9)	67 (6/9)
Candida lusitaniae	100 (5/5)	100 (5/5)	100 (5/5)	100 (5/5)	100 (5/5)	100 (5/5)	100 (5/5)	100 (5/5)
Total	90 (37/41)	93 (38/41)	73 (36/49)	82 (40/49)	91 (50/55)	91 (50/55)	76 (42/55)	78 (43/55)

a Strains with insufficient growth after 18 h are not included in the data analysis for this time point. Data for 36 h were calculated with the inclusion of 18 h data and additionally identified strains after 36 h. Data obtained with manual target reshooting are included in this table.

b Gray shading indicates the absence of data for *Candida glabrata* on HEM plates at 18 h.

Overall, after 18 h of incubation, 37/41 strains (90%) cultured on HEM plates were identified with an acceptable ID score when using Colibrí, compared to 38/41 strains (93%) when spot-plated manually. On the SAB plates, acceptable IDs were obtained in 73% (36/49) of the strains spot-plated using Colibrí and 82% (40/49) of the manually spot-plated strains. The cumulative percentage of acceptable IDs after 36 h of incubation for the strains spot-plated using Colibrí was 91% (50/55) for the HEM plates and 76% (42/55) for the SAB plates. For the manually spot-plated strains, the observed percentages were 91% (50/55) for the HEM plates and 78% (43/55) for the SAB plates. The differences between the percentages of acceptable IDs obtained using Colibrí and the manual method on HEM and SAB plates were not statistically significant, after either 18 h or 36 h of incubation.

**FIG 2:**
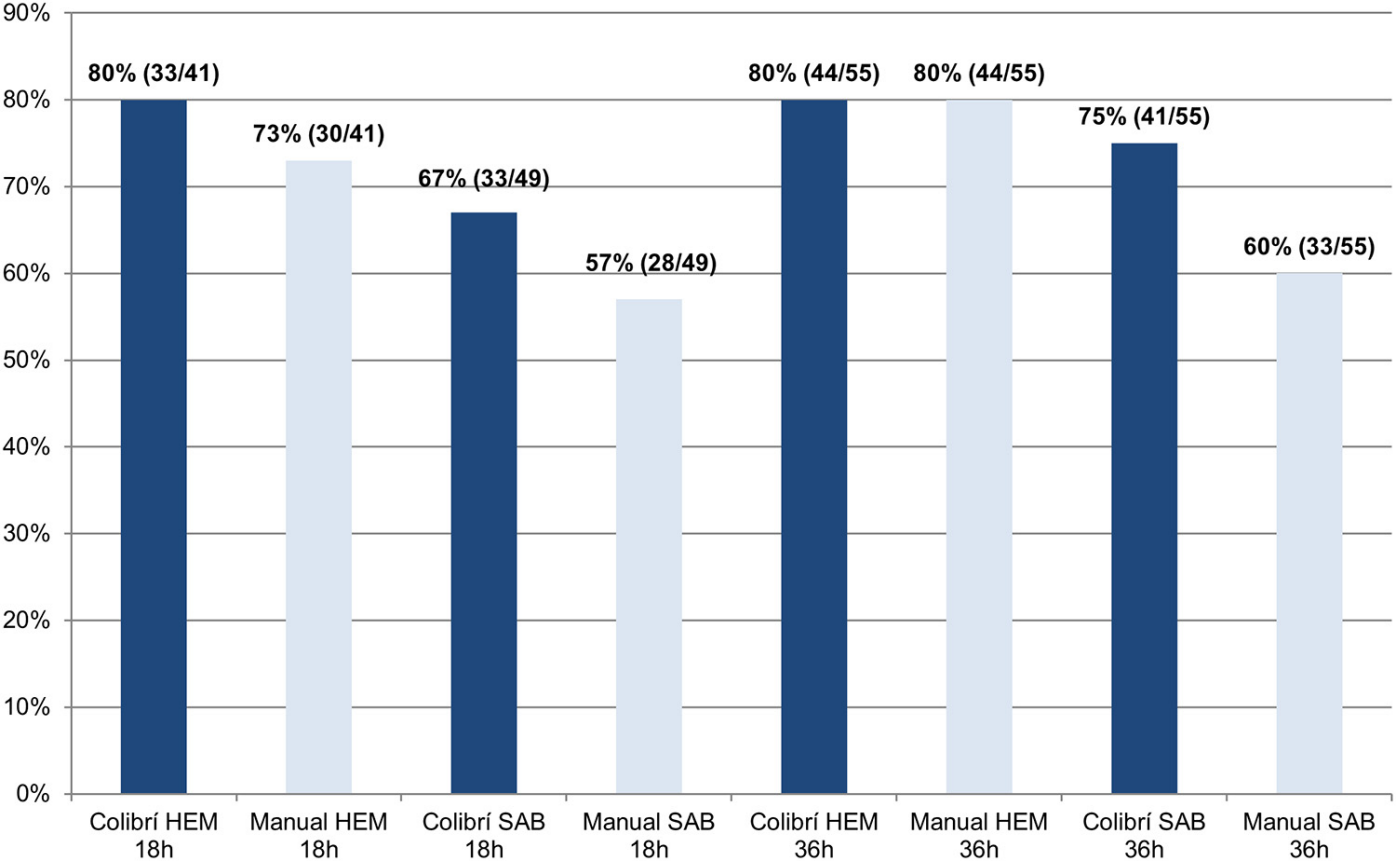
Acceptable identification after 18 h and 36 h of incubation (cumulative percentages), in automatic mode only. The graph shows the cumulative percentages of strains with an acceptable ID score after 18 h and 36 h of incubation. Strains with insufficient growth after 18 h are not included in the data analysis at this time point. The data for 36 h were calculated with the inclusion of 18 h data and additionally identified strains after 36 h. Only data obtained using the automatic mode of the MALDI Biotyper and thus without manual target reshooting are included in this graph.

An acceptable ID score was observed in 100% of strains spot-plated using Colibrí after 18 h of incubation for C. krusei (*n* = 2), C. auris (*n* = 2), C. guilliermondii (*n* = 6), and C. lusitaniae (*n* = 5) grown on both HEM and SAB plates, and for C. albicans (*n* = 6) and C. parapsilosis (*n* = 3) grown on HEM plates. For C. glabrata, growth was observed only on the SAB plates in 8/9 strains after 18 h of incubation, with acceptable IDs obtained in 88% of strains spot-plated using Colibrí and 63% of manually spot-plated strains. Fig. 2 shows the cumulative percentages of acceptable IDs after 18 h and 36 h of incubation if only automatic identification with MALDI-TOF (without manual reshooting of the target) was taken into account. Acceptable IDs on HEM plates were obtained for 80% of the strains spot-plated using Colibrí after both 18 h and 36 h of incubation. For the SAB plates, this percentage was lower, with 67% acceptable IDs for the strains spot-plated using Colibrí after 18 h and 75% after 36 h of incubation. Of note, when comparing the percentages of acceptable IDs obtained with and without manual target reshooting, the percentages of manually spot-plated strains identified at 18 h were significantly lower when no manual target reshooting was performed, both on the HEM and SAB plates (*P = *0.03 and *P = *0.01, respectively).

### Agreement between Colibrí and the manual method.

The agreement of identification was calculated between Colibrí and the manual method for the identifiable manually spot-plated strains. To calculate the cumulative agreement after 36 h of incubation, the agreement data at 18 h and the additionally identified strains after 36 h were included. Detailed results are provided in Table 2. The application of Colibrí did not cause any misidentification by MALDI-TOF. When 100% agreement between the automated and manual methods was not achieved, as was the case for example for C. dubliniensis and C. tropicalis strains, this was due to the absence of an identification with an acceptable ID score and not because of misidentification. Of the 38 *Candida* strains identified using the manual method on HEM plates after 18 h, 35 strains were also identified when using Colibrí, resulting in an overall agreement of 92% at this time point. After 36 h of incubation, the overall agreement for the HEM plates slightly increased to 94% (47/50), albeit this was not significantly different. Agreement of 100% was observed on the HEM plates after both 18 h and 36 h of incubation for C. albicans, C. krusei, C. auris, C. guilliermondii, C. parapsilosis, and C. lusitaniae strains. For the SAB plates, the overall agreement after 18 h was 78% (31/40). After 36 h of incubation, the overall agreement increased to 84% (36/43), albeit this was not significantly different from the agreement obtained after 18 h.

**TABLE 2:** Agreement between Colibrí and manual reference method after 18 h and 36 h of incubation (cumulative percentages)^*a*^

Species	% strains identified (no. identified with Colibrí/no. identified with manual method)			
	18 h		36 h	
	HEM	SAB	HEM	SAB
Candida albicans	100 (4/4)	83 (5/6)	100 (7/7)	83 (5/6)
Candida krusei	100 (2/2)	100 (2/2)	100 (2/2)	100 (2/2)
Candida auris	100 (2/2)	100 (2/2)	100 (2/2)	100 (2/2)
Candida dubliniensis	75 (6/8)	25 (1/4)	75 (6/8)	25 (1/4)
Candida guilliermondii	100 (6/6)	100 (6/6)	100 (6/6)	100 (6/6)
Candida parapsilosis	100 (3/3)	67 (2/3)	100 (7/7)	100 (5/5)
Candida tropicalis	88 (7/8)	57 (4/7)	88 (7/8)	57 (4/7)
Candida glabrata ^*b*^		80 (4/5)	100 (5/5)	100 (6/6)
Candida lusitaniae	100 (5/5)	100 (5/5)	100 (5/5)	100 (5/5)
Total	92 (35/38)	78 (31/40)	94 (47/50)	84 (36/43)

a Only identifiable manually spot-plated strains are included for calculation of agreement. Strains with insufficient growth after 18 h are not included in the data analysis for this time point. Cumulative data for 36 h were calculated with the inclusion of 18 h data and additionally identified strains after 36 h. Data obtained with manual target reshooting are included in this table.

b Gray shading indicates the absence of data for *Candida glabrata* on HEM plates at 18 h.

### Reproducibility.

For both the ATCC reference strains, C. albicans ATCC 90028 and C. glabrata ATCC 90030, a 100% within-run reproducibility (10/10 selected colonies identified) was observed on HEM and SAB plates when using Colibrí. For C. albicans ATCC 90028, we found a between-run reproducibility of 100% (10/10 selected colonies identified) on the HEM plates and 90% (9/10 selected colonies identified) on the SAB plates. When the reproducibility was not 100%, this was due to the absence of an identification with an acceptable ID score and not because of an incorrect ID. The C. glabrata strain (ATCC 90030) showed a between-run reproducibility of 90% (9/10 selected colonies identified) on the HEM plates and 100% (10/10 selected colonies identified) on the SAB plates. For the strains isolated from clinical samples that were used as well to test the reproducibility of Colibrí, C. albicans showed a within-run reproducibility of 100% for the HEM plates and 70% for the SAB plates. The C. glabrata strain showed a within-run reproducibility of 100% for both the HEM and SAB plates. The between-run reproducibility for both of the clinically derived strains was 100% for the HEM plates, with the C. albicans strain showing a 100% between-run reproducibility for the SAB plates as well. The clinically derived C. glabrata strain had a between-run reproducibility of 90% for the SAB plates. Colonies of the C. glabrata strains grown on HEM plates were identified after 36 h of incubation because of insufficient growth after 18 h.

## DISCUSSION

Correct identification of pathogens remains one of the most important tasks within the clinical microbiology laboratory. Our study has demonstrated that the Colibrí novel automated system can be used for preparation of MALDI-TOF target plates for yeasts in an accurate and reproducible way. However, there are certain observations that need to be addressed. The overall percentages of acceptable IDs for the manual method were lower after 36 h for both the HEM and SAB plates than after 18 h (Table 1). This apparent discrepancy in the results is caused by the fact that after 18 h, data for a limited number of strains were available because of the insufficient growth of 14 strains on the HEM plates and 6 strains on the SAB plates. In contrast, after 36 h, sufficient growth was available for all strains, but the number of additionally identified strains was proportionally smaller, hence causing a lower percentage of acceptable IDs. Inversely, when looking at the agreement results (Table 2) for the HEM plates, there was an increase from 92% to 94% overall agreement after 36 h. This was most likely due to the addition of the agreement data for C. glabrata (100% [5/5 strains]) after 36 h, which had not grown on HEM plates after 18 h, since the agreement results for the other included *Candida* species remained mostly unchanged. For the SAB plates, the increase in overall agreement after 36 h was even more pronounced (78% after 18 h versus 84% after 36 h). In particular, there was an increase in the agreement percentages for C. parapsilosis (67% [2/3 strains] after 18 h versus 100% [5/5 strains] after 36 h) and C. glabrata (80% [4/5 strains] after 18 h versus 100% [6/6 strains] after 36 h). The data obtained without manual target reshooting, as indicated in Fig. 2, show that Colibrí outperformed the manual method (especially on the SAB plates), although both Colibrí and the manual method generated 80% acceptable IDs (44/55 strains) at 36 h on the HEM plates. As can be expected, the overall percentages of acceptable IDs were lower without manual reshooting at all time points for both Colibrí and the manual method. However, these differences were most pronounced in the manually spot-plated strains, with the percentages of acceptable IDs being significantly lower on both the HEM and SAB plates after 18 h without manual target reshooting.

Nevertheless, all results obtained in this study at every time point, regardless of using the automatic acquisition mode or manual target reshooting of the MALDI-TOF, clearly indicate that strains are more difficult to identify when using SAB plates instead of HEM plates. However, C. glabrata showed no growth after 18 h on HEM plates, in contrast to the SAB plates, where 8/9 strains showed growth, and an acceptable ID was obtained in 88% of the strains spot-plated using Colibrí. Interestingly, in the two other *Candida* species associated with fluconazole resistance, C. krusei and C. auris, an acceptable ID percentage of 100% was observed on both plate types after 18 h of incubation. Reliable identification of those species in particular is important for guiding empirical therapy.

The suboptimal identification with MALDI-TOF of isolates cultured on SAB plates has previously been reported in the literature. Theel et al. and Van Herendael et al. obtained an identification with MALDI-TOF (with a cutoff log score of 1.7) in ± 80% of yeast isolates when directly spotting colonies grown on Sabouraud agar onto the MALDI-TOF target plate and performing an on-plate formic acid extraction (10, 11). Although these results are consistent with the identification percentages we observed on SAB plates when using the manual method, this is still considerably lower than the identification percentages of >90% observed in yeast isolates when using a tube-based protein extraction method (12, 13). Moreover, as was previously stated by Gorton et al., the reference spectra in the MALDI-TOF database are generated with conventional tube-based formic acid extraction and differ from the spectra obtained when using on-plate formic acid extraction, as we did in this study (14). Besides the impact of the culture plate type, this might also explain why we failed to identify certain *Candida* strains, both with the Colibrí and manual method. However, on-plate formic acid extraction is time saving and has the substantial advantage that it can be automated when using Colibrí, as is the case for the deposition of matrix solution.

One of the limitations of this study is that it was conducted on a limited number (55) of *Candida* strains. Confirmation of the observations made in this study on a larger sample cohort is highly desirable. However, this study was conducted from an explorative point of view, and based on the evidence we provided, Colibrí may be implemented into laboratory practice in laboratories worldwide for the identification of yeasts with MALDI-TOF, thus facilitating the validation of this device on a larger scale.

To conduct this study, we used a sheep blood agar plate supplemented with X and V factors (HEM plate), which is the default blood agar plate that is used in our laboratory and is made in-house. Although not commercially available, previous validation of this HEM plate for the identification of Gram-positive and Gram-negative bacteria, comparing the MALDI-TOF identification results with other, previously validated blood-supplemented agar plates (i.e., TSBA [tryptic soy blood agar], BLA [blood agar], and MHB [Mueller-Hinton blood]), showed a 100% agreement between the identification results obtained from the HEM plate and the other validated blood agar plates. Of note, as previously mentioned, Paolucci et al. reported 100% agreement between the identification results of Gram-negative bacteria grown on four different commercially available media when using Colibrí (8). In addition to this evidence for bacteria, for each of the *Candida* species included in this study, we tested one strain grown both on a HEM plate and a sheep blood agar (SBA) plate (Thermo Fisher Diagnostics) (data not shown) and compared the identification results. Based on this experiment, no discordant results between HEM and SBA plates were observed for strains spot-plated with Colibrí. These findings further support the transferability of the Colibrí performance results from HEM plates to other, commercially available blood agar plates.

In summary, our study is the first to demonstrate very good overall agreement between Colibrí and the manual reference method for identification of yeasts with MALDI-TOF MS, especially for isolates grown on blood-based culturing plates. Based on these results, Colibrí can be used to automate the preparation of MALDI-TOF target plates to ensure the reliable identification of yeasts. The connection between Colibrí and WASPLab makes this device suitable for inclusion into a total lab automation project. By reducing the hands-on time for laboratory technologists and ensuring more standardized sample treatment and traceability, Colibrí may prove to be an important advantage for clinical microbiology laboratories in this era of increasing automation.

## References

[1] Puig-Asensio M, Padilla B, Garnacho-Montero J, Zaragoza O, Aguado JM, Zaragoza R, Montejo M, Muñoz P, Ruiz-Camps I, Cuenca-Estrella M, Almirante B, REIPI. 2014. Epidemiology and predictive factors for early and late mortality in Candida bloodstream infections: a population-based surveillance in Spain. Clin Microbiol Infect 20:O245–O254. 10.1111/1469-0691.12380. 24125548

[2] Pappas PG, Lionakis MS, Arendrup MC, Ostrosky-Zeichner L, Kullberg BJ. 2018. Invasive candidiasis. Nat Rev Dis Primers 4:18026. 10.1038/nrdp.2018.26. 29749387

[3] Guinea J. 2014. Global trends in the distribution of Candida species causing candidemia. Clin Microbiol Infect 20 Suppl 6:5–10. 10.1111/1469-0691.12539. 24506442

[4] Desoubeaux G, Coste AT, Imbert C, Hennequin C. 2022. Overview about Candida auris: what's up 12 years after its first description? J Mycol Med 32:101248. 10.1016/j.mycmed.2022.101248. 35091280

[5] van Belkum A, Welker M, Pincus D, Charrier JP, Girard V. 2017. Matrix-assisted laser desorption ionization time-of-flight mass spectrometry in clinical microbiology: what are the current issues? Ann Lab Med 37:475–483. 10.3343/alm.2017.37.6.475. 28840984 PMC5587819

[6] Zimmermann S. 2021. Laboratory automation in the microbiology laboratory: an ongoing journey, not a tale? J Clin Microbiol 59:e02592-20. 10.1128/JCM.02592-20. 33361341 PMC8106703

[7] Bielli A, Lacchini C, Lombardi G, Grimaldi C, Vismara C. 2017. Colibrí^TM^: a new automatic system for colony picking and MALDI-TOF targets preparation. Abstr ASM Microbe 2017, New Orleans, LA. American Society for Microbiology, Washington, DC.

[8] Paolucci M, Navarria L, Castriciano S. 2019. Colibrí™ and Bruker MALDI-TOF: does the identification performance change when different chromogenic media are used for urine culture? Abstr 29th Eur Congr Clin Microbiol Infect Dis, Amsterdam, Netherlands.

[9] Copan. 2021. Colibrí operator manual. REV05. Copan, Brescia, Italy.

[10] Theel ES, Schmitt BH, Hall L, Cunningham SA, Walchak RC, Patel R, Wengenack NL. 2012. Formic acid-based direct, on-plate testing of yeast and Corynebacterium species by Bruker Biotyper matrix-assisted laser desorption ionization-time of flight mass spectrometry. J Clin Microbiol 50:3093–3095. 10.1128/JCM.01045-12. 22760034 PMC3421773

[11] Van Herendael BH, Bruynseels P, Bensaid M, Boekhout T, De Baere T, Surmont I, Mertens AH. 2012. Validation of a modified algorithm for the identification of yeast isolates using matrix-assisted laser desorption/ionisation time-of-flight mass spectrometry (MALDI-TOF MS). Eur J Clin Microbiol Infect Dis 31:841–848. 10.1007/s10096-011-1383-y. 21861205

[12] Marklein G, Josten M, Klanke U, Müller E, Horré R, Maier T, Wenzel T, Kostrzewa M, Bierbaum G, Hoerauf A, Sahl HG. 2009. Matrix-assisted laser desorption ionization-time of flight mass spectrometry for fast and reliable identification of clinical yeast isolates. J Clin Microbiol 47:2912–2917. 10.1128/JCM.00389-09. 19571014 PMC2738125

[13] Buchan BW, Ledeboer NA. 2013. Advances in identification of clinical yeast isolates by use of matrix-assisted laser desorption ionization-time of flight mass spectrometry. J Clin Microbiol 51:1359–1366. 10.1128/JCM.03105-12. 23426924 PMC3647935

[14] Gorton RL, Seaton S, Ramnarain P, McHugh TD, Kibbler CC. 2014. Evaluation of a short, on-plate formic acid extraction method for matrix-assisted laser desorption ionization-time of flight mass spectrometry-based identification of clinically relevant yeast isolates. J Clin Microbiol 52:1253–1255. 10.1128/JCM.03489-13. 24478407 PMC3993519

